# Atrial Myxoma in Both Chambers: Biatrial or Bilateral? A Rare Case Resected via Endoscopic Approach and Literature Review

**DOI:** 10.3390/medsci13040294

**Published:** 2025-11-30

**Authors:** Marius Mihai Harpa, Emanuel-David Anitei, Hussam Al Hussein, Mihaly Veres, Simona Gurzu, Diana Roxana Opris, Fiat Emilia Sorina, Emil Marian Arbanasi, Claudiu Ghiragosian, Cosmin Marian Banceu, Horatiu Suciu, Robert Balan

**Affiliations:** 1 Department of Surgery IV, George Emil Palade University of Medicine, Pharmacy, Science and Technology of Targu Mures, 38 Gheorghe Marinescu Street, 540139 Targu Mures, Romania; marius_harpa@yahoo.com (M.M.H.); claudiughiragosian@gmail.com (C.G.); cosmin.banceu@umfst.ro (C.M.B.); horisuciu@gmail.com (H.S.); 2 Doctoral School of Medicine and Pharmacy, George Emil Palade University of Medicine, Pharmacy, Sciences and Technology of Targu Mures, 540142 Targu Mures, Romania; v_misy@yahoo.com (M.V.); dianaroxana.opris@yahoo.com (D.R.O.); emil.arbanasi@umfst.ro (E.M.A.); balanrobert2003@gmail.com (R.B.); 3 Department of Cardiac Surgery, Emergency Institute for Cardiovascular Diseases and Transplantation of Targu Mures, 540136 Targu Mures, Romania; 4 Department of Anatomy and Embriology, George Emil Palade University of Medicine, Pharmacy, Science and Technology of Targu Mures, 540139 Targu Mures, Romania; alhussein.hussam@yahoo.com; 5 Department of Anesthesia and Intensive Care, Emergency Institute for Cardiovascular Diseases and Transplantation of Targu Mures, 540136 Targu Mures, Romania; 6 Department of Pathology, George Emil Palade University of Medicine, Pharmacy, Science, and Technology of Targu Mures, 540139 Targu Mures, Romania; simonagurzu@yahoo.com; 7 Department of Cardiology, Emergency Institute for Cardiovascular Diseases and Transplantation of Targu Mures, 540136 Targu Mures, Romania; 8 Department of Cardiology, Emergency Clinical Hospital, 550245 Sibiu, Romania; sorina.fiat@yahoo.com; 9 Department of Cardiac Surgery, Klinikum Passau, 94032 Passau, Germany

**Keywords:** biatrial myxoma, bilateral myxoma, endoscopic cardiac surgery, cardiac tumor

## Abstract

Background: Primary cardiac tumors are exceedingly rare, with myxomas representing the most common benign type among these tumors, predominantly located in the left atrium. Biatrial involvement is an exceptional presentation, reported in less than 2.5% of cases. The terms bilateral and biatrial atrial myxoma are often confused and used interchangeably. We present a rare case of atrial myxoma involving both chambers, resected via a minimally invasive endoscopic approach, accompanied by a literature review. Case Presentation: A 52-year-old male with a three-month history of progressive fatigue and exertional dyspnea was found, on transthoracic echocardiography and cardiac MR, to have two intracardiac masses affecting both atria. Intraoperative transesophageal echocardiography confirmed the presence of mirror-image myxomas attached to the interatrial septum, in the absence of any septal defect. The tumors were excised en bloc, including the septal attachment, using a minimally invasive endoscopic approach. Histopathological examination confirmed the diagnosis of atrial myxoma, and the postoperative course was uneventful. Conclusions: Bilateral and biatrial atrial myxomas are exceedingly rare forms of cardiac tumors. In many cases, the first clinical manifestations may arise from cerebral or peripheral embolic events or from pulmonary thromboembolism. For this reason, screening echocardiography may be useful, particularly in cases of familial myxoma. Transthoracic and transesophageal echocardiography, combined with careful inspection of all cardiac chambers, play a crucial role in preventing recurrence by detecting small or overlooked tumor fragments. The minimally invasive endoscopic approach offers a safe and effective surgical option for biatrial myxomas, providing excellent visualization and facilitating thorough exploration of the cardiac cavities.

## 1. Introduction

The first surgical excision of a bilateral atrial myxoma was reported in 1967 by Tada, Yipintsoi et al. [1]. Primary cardiac tumors are extremely rare, with an incidence of 0.0017–0.03%, and approximately 75% are benign, half of which are myxomas. Most myxomas arise in the left atrium (83%), followed by the right atrium (12.7%), whereas biatrial involvement is exceptional, with a reported incidence below 2.5% [2]. Although often used interchangeably, the terms biatrial and bilateral myxoma may describe different entities. Bilateral myxomas represent two independent tumors originating in each atrium, while true biatrial myxomas consist of a single mass crossing the interatrial septum through a patent foramen ovale (PFO) or atrial septal defect (ASD).

Myxomas can be solitary or multiple and may arise in various cardiac locations, including the atria, ventricles, great vessels, valves, or septal structures [3]. Histologically, they are broadly categorized as solid or papillary, the latter having higher embolic potential due to their friable consistency [4,5].

Bilateral myxomas may occur sporadically or in association with Carney complex (CNC), an autosomal dominant syndrome characterized by PRKAR1A mutations, multiple myxomas, endocrine tumors, and cutaneous pigmentation [6]. Up to 7% of myxomas occur in CNC, where recurrence rates after resection are significantly higher (up to 44%) compared with sporadic cases (1–3%) [7]. Clinical manifestations are diverse and often nonspecific, typically involving obstructive symptoms, embolic events, or constitutional manifestations such as fever and fatigue [8]. Embolic phenomena may be the initial presentation, particularly in friable or villous tumors [9]. Diagnosis relies on multimodal imaging, with transesophageal echocardiography (TEE) providing near-100% sensitivity and essential information about tumor origin and attachment [10].

Surgical resection remains the gold standard to prevent complications and recurrence. While median sternotomy is the traditional approach, minimally invasive endoscopic surgery has shown promising results in selected patients, offering excellent visualization and reduced morbidity [11]. Intraoperative TEE, comprehensive exploration of all chambers, and en bloc tumor excision with safe margins are recommended to minimize recurrence [12].

Here, we describe a rare case of mirror-image bilateral atrial myxomas successfully excised via a minimally invasive endoscopic approach and present a comprehensive systematic review of published cases, focusing on clinical presentation, imaging characteristics, tumor morphology, surgical management, and outcomes.

## 2. Case Presentation

**Figure 1:**
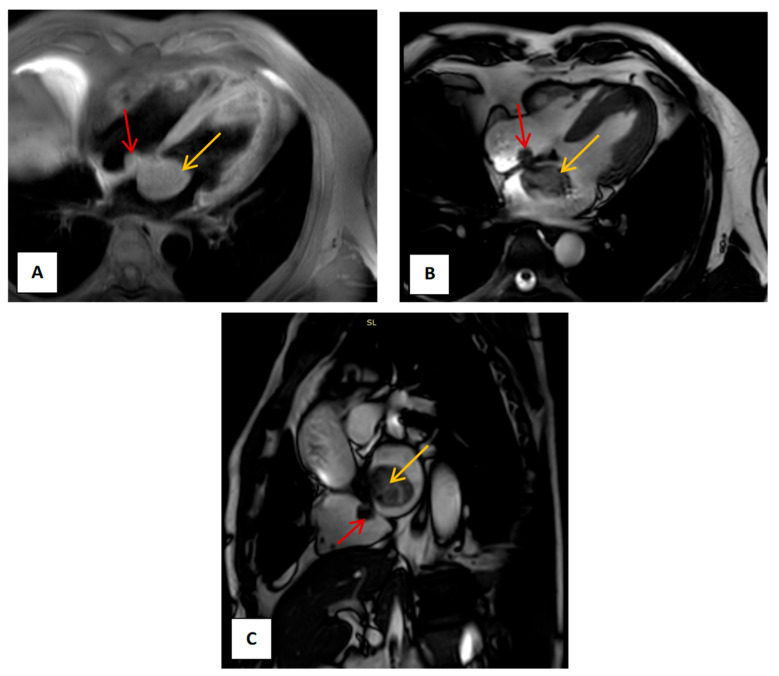
Cardiac MR: (**A**) four-chamber view, T1 sequence. The yellow arrow indicates the tumor located in the left atrium, while the red arrow highlights the tumor arising from the right atrium. (**B**) four-chamber view, using a balanced steady-state free precession (bSSFP) sequence. The intracardiac masses are visualized attached to the interatrial septum, which appears intact. (**C**) sagittal view, using a bSSFP, showing both tumors.

We present the case of a 52-year-old male patient who reported fatigue and exertional intolerance for the past three months. Transthoracic echocardiography revealed a tumoral mass involving both atria. The patient was referred to our center for further evaluation. Clinically, he complained of dyspnea on moderate exertion, while laboratory tests showed no pathological findings. A thoraco-abdomino-pelvic CT scan identified two intracardiac masses located in both atria, with no signs of pulmonary thromboembolism, cerebral, or peripheral embolism. Coronary angiography revealed no significant stenoses. Cardiac magnetic resonance (MR) imaging confirmed the presence of two masses attached to the interatrial septum, measuring 4 × 3 cm in the left atrium and 2 × 1.5 cm in the right atrium (Figure 1).

**Figure 2:**
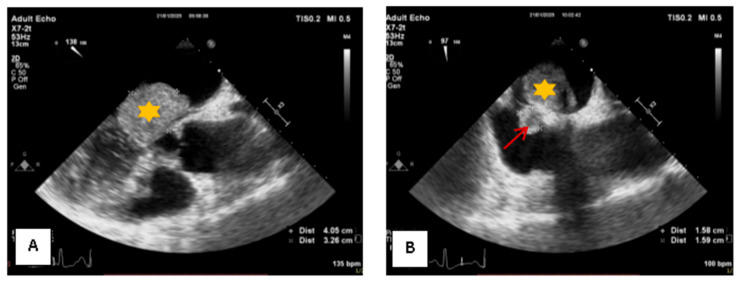
Transesophageal Echocardiography: (**A**) The left atrial tumor, measuring 4.0 × 3.2 cm, is marked with a yellow asterisk. (**B**) The right atrial tumor, measuring 1.5 × 1.5 cm, is marked with a red arrow.

Following discussion within the Heart Team and obtaining informed consent, the decision was made to proceed with surgical excision via a minimally invasive endoscopic approach. Intraoperative transesophageal echocardiography revealed two tumor masses attached to the interatrial septum with mirror-like implantation sites measuring 4.0 × 3.2 cm on the left side and 1.5 × 1.5 cm on the right without evidence of interatrial communication (Figure 2).

**Figure 3:**
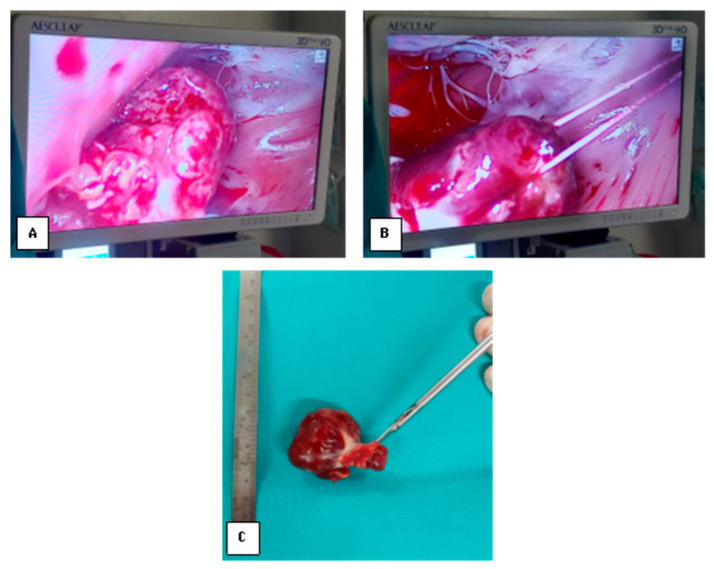
(**A**) Globular aspect of the left atrial myxoma. (**B**) The left atrial myxoma is secured with a guiding suture; the mitral valve is visible on the right side of the image. (**C**) En bloc view of the biatrial myxoma.

The surgical approach followed the standard endoscopic protocol, including peripheral cannulation (right femoral artery and jugulo-femoral venous cannulation), a 3.5 cm working incision in the fourth intercostal space, a 3D endoscopic camera inserted in the second intercostal space, and aortic clamp via the third intercostal space. The left atrium was opened, revealing a solid, oval-shaped tumor with firm consistency, originating from a broad base on the interatrial septum. The mass was excised en bloc, including the involved segment of the interatrial septum and the right atrial tumor, which originated in a mirrored location on the septum (Figure 3).

**Figure 4:**
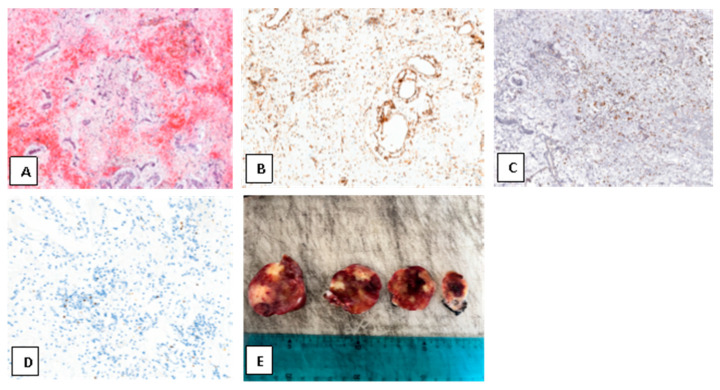
(**A**) Hematoxylin and eosin staining (magnification 10×) showing the typical myxoid stroma with prominent vascularization and areas of hemorrhagic extravasation within the atrial myxoma. (**B**) Immunohistochemical staining for CD31 (magnification 20×), highlighting the rich vascular network within the atrial myxoma. (**C**) Immunohistochemical staining for CD68 (magnification 20×), showing scattered histiocyte/macrophage infiltration within the myxoid stroma of the atrial myxoma. (**D**) Immunohistochemical staining for Ki-67 (magnification 40×), showing low proliferative activity, consistent with the benign nature of atrial myxoma. (**E**) Gross pathology shows sectioned tumor fragments with a lobulated, gelatinous texture and heterogeneous coloration, including areas of hemorrhage.

The septum was reconstructed using a continuous suture. Histopathological examination confirmed the diagnosis of myxoma (Figure 4).

The postoperative course was uneventful. Follow-up transthoracic echocardiography at 8 months revealed normal cardiac function with no evidence of recurrence.

## 3. Materials and Methods

**Figure 5:**
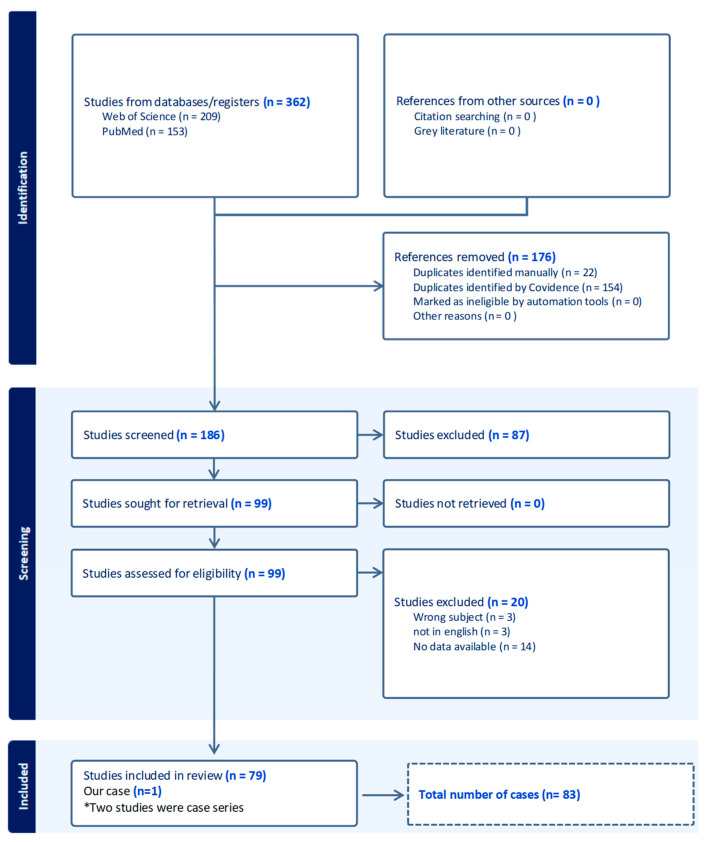
PRISMA 2020 protocol.

A comprehensive literature review was conducted using two electronic databases, PubMed and Web of Science, in accordance with the PRISMA 2020 guidelines (Figure 5). The review was not eligible for PROSPERO registration because data extraction had already begun. The search terms used were “biatrial cardiac myxoma” and “biatrial myxoma.” Only human studies were considered, with no restrictions on patient age or date of publication. Both case reports and case series were eligible for inclusion. In instances where case reports were also cited within review articles, only the original case report was retained to avoid duplication. Review-only articles were excluded from the analysis. All references were imported into Covidence (Veritas Health Innovation, Melbourne, Australia) for screening, deduplication, record management, workflow automation, and independent assessment by two reviewers of all titles, abstracts, and full-text articles according to predefined inclusion criteria, with any disagreements resolved through discussion and no additional automation tools used. The initial search yielded 362 articles. After removing duplicates and applying the inclusion and exclusion criteria (non-English language, animal studies, lack of relevant data, or unrelated subjects), 79 studies were retained, among which two were case series. This process resulted in a final dataset of 83 analyzed cases, one of which is the case we report. For each case, data were extracted regarding demographic and clinical characteristics, including author, title, year of publication, patient sex and age, presenting symptoms, family history of cardiac tumors, and whether the patient had previously undergone reoperation for recurrence. Tumor-related details included the presence or absence of interatrial communication, such as atrial septal defect or patent foramen ovale, associated conditions, presence of arrhythmias, tumor dimensions in both atria, implantation sites, and whether the tumors had distinct origins. Imaging modalities used in diagnosis were also recorded, including transthoracic and transesophageal echocardiography, computed tomography, cardiac magnetic resonance imaging, and coronary angiography or catheterization. Surgical information was collected regarding the type of procedure performed, the atrial approach, the surgical access route, whether by sternotomy, thoracotomy, or minimally invasive endoscopic technique, and any postoperative complications. Additional data are summarized in Table S1 (Supplementary Materials). Descriptive statistics were used for analysis, with categorical variables presented as absolute numbers and percentages. The study was conducted in accordance with the Declaration of Helsinki and approved by the Ethics Committee of Emergency Institute for Cardiovascular Diseases and Transplantation, Targu Mures, Romania (protocol no. 5636; date of approval: 20 October 2025).Written informed consent has been obtained from the patient to publish this paper.

## 4. Results

**Figure 6:**
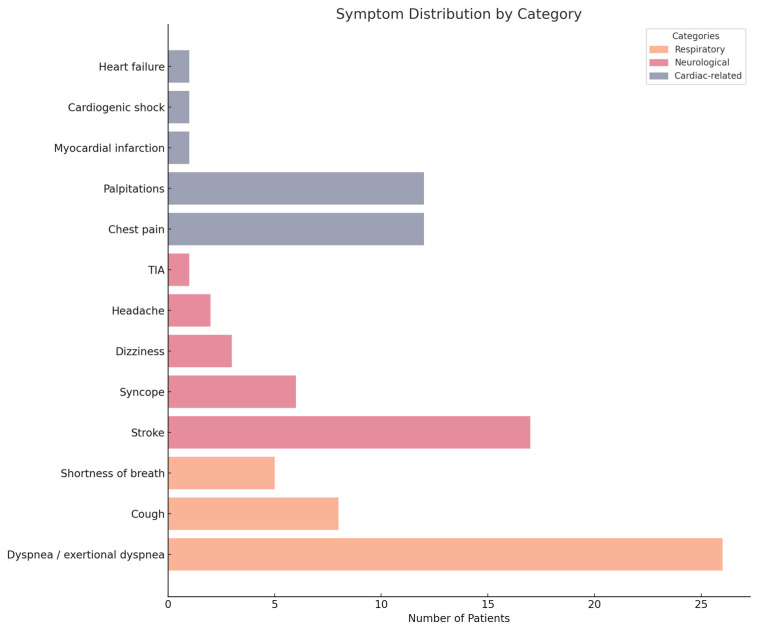
Symptom distribution by category.

Three hundred sixty-two articles were initially identified through systematic searches in Web of Science and PubMed. Eighty-three cases were included in the final analysis. Patient age ranged from 2 months to 79 years. Among the included cases, 9 patients (10.8%) were younger than 18 years, 73 patients (87.9%) were adults aged 18 years or older, and in 1 case (1.2%), the age was not reported. The mean age was 10.4 ± 5.6 years for pediatric patients and 45.1 ± 15.5 years for adults. Of the 83 cases, 50 were female (60.2%), and 33 were male (39.8%). Fourteen patients (16.8%) were asymptomatic at presentation. In two cases (2.4%), symptom information was not available, and in one case (1.2%), the patient was described as symptomatic without further specification. The most frequently reported symptoms were respiratory, present in 45 patients (54.2%), followed by neurological symptoms in 32 patients (38.6%). Among the respiratory manifestations, dyspnea or exertional dyspnea was the most common, occurring in 26 patients (31.3%), followed by cough in 8 patients (9.6%), shortness of breath in 5 patients (6%), and (less frequently) hemoptysis (3 patients, 3.6%). Neurological symptoms included stroke in 17 patients (20.5%), syncope in 6 (7.2%), dizziness in 3 (3.6%), and headache in 2 (2.4%). Transient ischemic attack (TIA) was documented in 1 patient (1.2%). Cardiac-related manifestations were also frequent, with chest pain and palpitations reported in 12 patients each (14.5%). Additionally, myocardial infarction, cardiogenic shock, and heart failure were each identified in 1 patient (1.2%). Systemic or general symptoms were also common. Fatigability was reported in 10 patients (12%), while fever occurred in 9 patients (10.8%). Peripheral edema or lower-extremity swelling was noted in 4 patients (4.8%). For an easier visualization of the most frequent symptoms, please refer to Figure 6: Symptom distribution by category.

Sixty-one patients (73.5%) presented with sporadic myxomas, while 21 cases (25.3%) were classified as familial. Among these, 11 patients (13.3%) were explicitly associated with Carney complex. Regarding reintervention for recurrence, 12% of cases underwent repeat surgery for recurrent myxomas, while in 2.4% the information was not specified. More than 80% of patients did not present any interatrial septal communication, whereas 5 patients (6%) had an atrial septal defect (ASD) and 7 patients (8.4%) had a patent foramen ovale (PFO). Regarding associated valvular pathologies, 13 patients (14.5%) had mitral regurgitation, of which 5 cases were moderate, and 1 case was severe. Mitral stenosis was found in 3 patients (3.6%). Tricuspid regurgitation occurred in 9 patients (10.8%), including 3 moderate and 1 severe case. Additionally, tricuspid stenosis was identified in 3 patients (3.6%). Anemia was reported in 10 patients (12%), diabetes mellitus in 4 patients (4.8%), and hypertension in 5 patients (6%). Pulmonary embolism was noted in 9 cases (10.8%), while 1 patient (1.2%) had pulmonary infarction and 1 patient (1.2%) presented with pneumonia. Additional respiratory-related conditions included asthma (1.2%) and pleural–pericardial effusion (2.4%). Cardiovascular complications included embolic occlusion of the left anterior descending artery (1.2%), stenosis of the same artery (1.2%), angina due to a coronary steal phenomenon (1.2%), interatrial septal aneurysm (1.2%), and endocarditis in 1 patient (1.2%). Endocrine and neoplastic disorders were diverse and included: ovarian cyst (1.2%), bilateral oophorectomy (1.2%), primary pigmented nodular adrenocortical disease (2.4%), Cushing’s syndrome (2.4%), acromegaly (1.2%), breast tumors (3.6%), mastectomy for neoplasia (1.2%), Sertoli cell tumor of the testis (2.4%), hypophyseal adenoma (2.4%), pituitary tumor (1.2%), colonic tubular adenoma (1.2%), pheochromocytoma (1.2%), inguinal superficial angiomyxoma (1.2%), neurofibroma (2.4%), bilateral adrenalectomy (2.4%), spinal melanotic schwannoma (1.2%), thyroid nodules (2.4%), Raynaud’s syndrome (1.2%), Leiden factor V mutation (1.2%).

Regarding rhythm disturbances, atrial fibrillation occurred in 7 patients (8.4%), and atrial flutter in 3 patients (3.6%). One patient (1.2%) presented with left bundle branch block. Most patients (84.3%) had no documented rhythm abnormalities. Tumor sizes varied considerably, with the largest lesion measuring 13.7 × 9.7 cm and the smallest 0.4 × 0.4 cm. In 10 cases, the site of myxoma attachment was not specified. Two cases were described only as multiple tumors. Tumors were categorized according to their location in the left or right atrium. When no interatrial communication (ASD or PFO) was identified by imaging, intraoperative assessment, or histopathology, and when the tumors originated from distinct sites, the lesions were classified as bilateral myxomas. In contrast, when an interatrial communication (ASD or PFO) was present, and both tumors shared a common origin, they were classified as single biatrial myxomas. A total of 12 cases had documented interatrial communication: 5 with atrial septal defect and 7 with patent foramen ovale. One PFO case was excluded from the single-biatrial group because the patient had multiple tumors. Thus, 11 cases were confirmed as single-origin biatrial myxomas. In 69 cases (83.1%), no interatrial communication was identified by imaging, intraoperative findings, or histopathology. When including the PFO case with multiple tumors, a total of 70 cases were classified as bilateral myxomas. In 2 cases, the available data were insufficient to determine the classification. All single biatrial myxomas shared a common origin: 9 originated from the interatrial septum (IAS), 1 from the ASD, and 1 case lacked explicit information regarding the origin. Among the 61 cases of bilateral myxomas, at least one tumor was attached to the interatrial septum. Of these, 45 cases (55.3%) had tumors arising from the same septal level, presenting as mirror-image myxomas.

Transthoracic echocardiography (TTE) was performed in 90.3% of cases, while transesophageal echocardiography (TEE) was used in 48.1%. Computed tomography (CT) was utilized in 32.5%, and cardiac magnetic resonance imaging (MRI) in 10.8% of patients. Surgical excision was performed in 80 patients (96.4%). In 2 cases (2.4%), no information regarding surgical management was available, and 1 patient (1.2%) died before undergoing surgery. Ablation at the tumor origin site was reported in 2 cases (2.4%), with one patient undergoing cryoablation and the other radioablation. Tricuspid valve repair was performed in 3 patients (3.6%), mitral valve repair in 2 patients (2.4%), and mitral valve replacement in 1 patient (1.2%). The majority of patients underwent surgical excision through a right atrial approach (37 patients; 44.5%). A biatrial approach was used in 14 cases (16.8%), while a left atrial approach was performed in 4 cases (4.8%). In 25 cases (30.1%), the surgical approach was not specified. Additionally, 1 patient (1.2%) underwent tumor excision via a superior atrial septal approach, and 1 patient (1.2%) had complete excision and reconstruction of both atria. Most patients underwent surgery via median sternotomy (49 patients; 59%). Additionally, 1 patient (1.2%) was operated through ministernotomy, 1 patient (1.2%) via thoracotomy, and 2 patients (2.4%) through a minimally invasive endoscopic approach.

Postoperative outcomes included the need for permanent pacemaker implantation in 4 patients (4.8%). Transient postoperative atrial fibrillation occurred in 2 patients (2.4%), and residual tumor tissue was identified in 2 patients (2.4%). One patient (1.2%) died before surgery, and another (1.2%) experienced early postoperative death due to massive systemic embolism. Additionally, 1 patient (1.2%) developed hypostatic pneumonia.

## 5. Discussion

The first successful surgical resection of a cardiac myxoma was performed by Clarence Crafoord in 1954, as highlighted by Chitwood in 1992 [13]. Interestingly, the history of cardiac tumor surgery can be traced even further, with the first recorded excision of a cardiac tumor attributed to Columbus of Padua in 1559 [14]. Cardiac myxomas can occur across all age groups, with peak incidence in the general population typically observed between the third and sixth decades of life [15]. In women, however, the highest frequency is reported between the ages of 50 and 60 years. Epidemiological data indicate that females are approximately twice as likely to develop this condition compared to males. Most myxomas originate in the left atrium (83%), followed by the right atrium (12.7%), while biatrial involvement is exceedingly rare, with some studies reporting an incidence ranging from 2.5% to 4%; other reports indicate an incidence as low as 1.3% of cases. In our cohort, the mean age among adult patients was 45.1 ± 15.5 years, and the gender distribution aligned with the literature, with a predominance of female patients (60.2%) [2,6,16]. Myxomas account for 21.5% of benign cardiac tumors in children, yet biatrial forms are extremely rare and poorly described in the literature. Ran et al. [17] reported only six pediatric cases, while our study identified nine. Gene expression data suggest that cardiac myxomas may originate from primitive, multipotent mesenchymal cells resembling the primordial cardiac stem cells of the fetal endocardium. These findings raise the possibility that remnants of embryonic cardiac tissue persist as islands of precursor cells capable of later tumorigenic proliferation, explaining the development of simultaneous myxomas from multiple pretumorous foci [18]. Familial clustering of cardiac myxomas has been recognized since the early 1970s, with Krause et al. [19] reporting the first familial cases in siblings. Myxomas are generally classified as sporadic or familial, with the majority being sporadic, consistent with our findings, in which over 70% of the cases were sporadic. Among the familial cases, a proportion is associated with Carney complex (CNC), a hereditary syndrome characterized by multiple cardiac and extracardiac myxomas [20,21]. In our review, more than 25% of cases had familial transmission, and 13.3% were diagnosed with CNC, including two pediatric patients. In a cohort of 319 CNC patients, Pitsava et al. [7] reported that 42.6% developed at least one cardiac myxoma, most commonly in the left atrium, while biatrial involvement remained rare but clinically relevant due to its potential for embolization, obstruction, and recurrence. Excess growth hormone has also been associated with an increased risk of recurrence, suggesting a multifactorial pathogenesis [22]. In our case, no clinical or genetic features suggestive of CNC were identified, supporting the likelihood of a sporadic occurrence. Recurrence of cardiac myxomas is an important clinical concern and may result from residual tumor tissue, multicentric growth, familial predisposition, or embolic implantation [23]. Overall recurrence rates range between 1 and 3% in the general population but are significantly higher in patients with CNC, reaching 10–44%. Demographic patterns have also been observed, with a higher recurrence tendency in younger individuals and a marked female predominance, who are reported to be 2.5 times more likely to develop new tumors [24]. In our review, 10 patients (11.1%) experienced recurrence, of whom 90% had familial transmission, and 10% were sporadic. Among these recurrent cases, five patients (50%) had CNC (one pediatric and four adult cases). The majority were adults with a mean age of 42.7 ± 10.2 years. Our findings align with previously published data, confirming that recurrence occurs predominantly in familial cases and more frequently in younger patients, with a strong female predominance. The classification proposed by Vega et al. [25] offers a valuable framework for understanding the complex morphology of atrial myxomas. This system includes four distinct patterns: Type A, biatrial myxoma with independent stalks; Type B, biatrial myxoma sharing a common stalk (mirror myxoma); Type C, multiple independent lesions; and Type D, a single myxoma traversing an atrial septal defect, representing the true biatrial form. In the context of our analysis, this classification was adapted to more clearly differentiate between the terms biatrial and bilateral. From a clinical and pathological perspective, atrial myxomas can be categorized based on both their number and location within the cardiac chambers. Most cases are solitary, but multiple tumors may occasionally occur, either within the same atrium or involving both. According to their distribution, three main patterns are described: uniatrial, biatrial, and bilateral myxomas. Uniatrial myxomas are confined to a single atrium, most commonly the left, and represent the typical presentation. Biatrial myxomas consist of a single tumor with one site of origin that extends through an interatrial communication such as a patent foramen ovale or an atrial septal defect, occupying both atria simultaneously. In contrast, bilateral myxomas are composed of two or more independent tumors that arise separately in the right and left atrium, without any communication or structural connection through the interatrial septum. Approximately 75% of biatrial myxomas exhibit a dumbbell or butterfly-shaped morphology, with two pedicles connected through the fossa ovalis, while cases involving distinct, non-continuous pedicles are exceedingly rare. In contrast, bilateral myxomas with clearly separate and non-homologous origins can be readily classified based on their independent attachment sites in each atrium.

However, classification becomes more complex in cases of homologous or “mirror” myxomas, where both tumors originate from symmetrical locations on opposite sides of the interatrial septum. In such situations, the absence of a detectable interatrial communication either on imaging or intraoperatively makes it challenging to determine whether the lesion represents a true biatrial tumor or two bilateral, independent formations. A definitive distinction requires evidence of a transseptal connection or macroscopic continuity between the tumors, as observed in the case reported by Li Yanhui et al. [26], where the masses were connected through the fossa ovalis. In ambiguous cases, histopathological examination remains essential for confirming whether the lesions share a common stalk and tissue continuity or represent separate neoplastic origins. In the presented patient, the myxoma originated bilaterally with mirror-image attachment sites, without any evidence of interatrial septal communication, and was therefore classified as bilateral. In our study, a total of 70 cases were identified as bilateral myxomas, of which 45 cases (55.3%) exhibited tumor origins at the same septal level, presenting as mirror-image myxomas. Additionally, 11 cases were confirmed as single-origin biatrial myxomas. It is noteworthy that 55.3% of bilateral myxomas demonstrated homologous, mirror-like origins without visible septal communication. In these instances, histopathological examination would have been crucial to determine whether the lesions shared a common origin or represented distinct tumors. This observation underscores the need for further detailed studies. Several studies have demonstrated a statistically significant association between tumor size and clinical presentation, showing that larger atrial myxomas (>5 cm) are more frequently linked to cardiac symptoms, yet paradoxically tend to remain undiagnosed for a longer period after symptom onset, possibly due to their slow growth and nonspecific manifestations. Reported estimates of left atrial myxoma growth rates range from 1.8 to 5.7 cm per year [27,28]. The clinical presentation of cardiac myxomas is highly variable and primarily depends on the tumor’s size, location, and mobility, although a subset of patients may remain asymptomatic. In our study, 16.8% of cases were incidentally discovered in asymptomatic individuals. Despite their benign histology, myxomas can produce serious complications such as hemodynamic obstruction, embolization, and systemic manifestations. In cases of biatrial or bilateral myxomas, the risks are combined due to the involvement of both systemic and pulmonary circulatory systems. These patients may experience simultaneous systemic embolization, including cerebral, peripheral, or visceral emboli, and pulmonary embolism, as described in the case reported by Ran Haifeng et al. [17]. This dual embolic potential underscores the complexity and severity of these rare tumor configurations. Morphological characteristics influence the risk of embolization. Polypoid myxomas are typically compact and less prone to spontaneous fragmentation, though embolic events may still occur depending on their mobility. In contrast, villous or papillary myxomas possess fine, gelatinous, and friable extensions that easily detach, making them significantly more emboligenic [29]. Hemodynamic symptoms result from obstruction of blood flow. Left atrial myxomas often cause dyspnea, chest discomfort, or hemoptysis, while right atrial tumors may produce edema, hepatomegaly, or ascites. Large masses can obstruct the atrioventricular valve, leading to syncope, pulmonary hypertension, or even sudden death. Approximately 20–45% of patients with cardiac myxomas experience systemic embolization, most frequently involving the cerebral circulation, but also the peripheral arteries, mesenteric vessels, and, more rarely, the coronary arteries. In our study, the second most frequent class of manifestations was neurological, occurring in 38.6% of patients. Among these, 17 patients (20.5%) presented with stroke as the initial manifestation, and 1 patient (1.2%) experienced TIA [30]. Embolic events are common due to the tumor’s friable texture. Left atrial lesions may lead to cerebral or peripheral emboli, whereas right atrial tumors can cause pulmonary embolism. Direct coronary artery involvement is exceptionally rare. However, in our series, one patient presented with occlusion of the left anterior descending (LAD) artery due to embolization, similar to the case described by Gagan Jain et al. [31]. Furthermore, a coronary steal phenomenon has been reported in the literature in a patient with giant bilateral myxomas exhibiting dual coronary vascularization, emphasizing the potential for complex hemodynamic effects in such cases [32]. In terms of clinical presentation, the most common manifestations in our cohort were respiratory symptoms, observed in 54.2% of patients. Among these, dyspnea or exertional dyspnea was the most frequent, recorded in 26 patients (31.3%), followed by cough in 8 patients (9.6%), and shortness of breath in 5 patients (6%). Hemoptysis was less common, seen in 3 cases (3.6%). It is worth noting that in biatrial or bilateral myxomas, distinguishing whether dyspnea is primarily of cardiac or respiratory origin can be challenging, as both circulations are affected. In severe cases, pulmonary hypertension may develop, and large tumors can obstruct the atrioventricular valve orifice, potentially leading to syncope or sudden death. Regarding arrhythmias, no cases of atrioventricular block were observed preoperatively in our cohort. However, atrial fibrillation occurred in 7 patients (8.4%), atrial flutter in 3 patients (3.6%), and one patient (1.2%) presented with a left bundle branch block [33]. Systemic and non-specific symptoms are thought to arise from interleukin-6 (IL-6)–mediated inflammatory responses induced by the tumor, including fever, fatigue, weight loss, arthralgia, myalgia, and anemia. Excess IL-6 contributes to systemic inflammation and may play a role in cardiac dysfunction [34]. In our study, fatigability was observed in 10 patients (12%), fever in 9 (10.8%), and anemia in 10 patients (12%). In addition to cardiac manifestations, patients with Carney complex may develop a wide range of extracardiac features involving the skin, endocrine glands, and nervous system. The most frequent findings include pigmented skin lesions, cutaneous and mammary myxomas, as well as endocrine disorders such as adrenal cortical disease and pituitary adenoma. Other reported manifestations are melanotic schwannomas, thyroid abnormalities, and testicular tumors in male patients [35]. Our patient presented with exertional dyspnea as the only symptom. Echocardiography showed that the myxoma did not cause obstruction or dysfunction of either the mitral or tricuspid valve, and there was no underlying pulmonary disease. Furthermore, CT evaluation revealed no signs of pulmonary thromboembolism. Although dyspnea was present, we cannot conclusively determine a direct causal relationship between this symptom and the presence of the tumor. Dyspnea may occur even in the absence of significant hemodynamic obstruction, potentially due to intermittent flow limitation, subtle diastolic impairment, or anxiety related to the cardiac mass, although these mechanisms remain speculative in this case. Although echocardiography remains the first-line diagnostic tool, it may have limitations, even in experienced hands, when distinguishing myxomas from other cardiac tumors or masses that can mimic similar echocardiographic characteristics. Transthoracic echocardiography was diagnostic in 95.2% of cases, while transesophageal echocardiography achieved 100% diagnostic accuracy. CT or MRI identified the lesion in 70% of cases. The site of tumor attachment was correctly identified in 64.5% by transthoracic and in 95.2% by transesophageal echocardiography [10,36]. Coronary angiography has proven useful in identifying feeding arteries, often arising from the right coronary artery. In addition, coronary CT angiography and three-dimensional reconstructions play an important role in evaluating tumor vascularization, which can be substantial in some cases, as reports describe myxomas with significant independent blood supply [37]. The treatment of cardiac myxomas is mainly surgical, as recommended by current cardio-oncology guidelines, due to the risk of embolization, obstruction, or sudden death. The surgical goal is to achieve complete excision of the tumor and its attachment site to prevent recurrence, while preserving cardiac structures and function. Intraoperative transesophageal echocardiography (TEE) is essential for confirming complete resection and assessing cardiac performance immediately after surgery [38]. The choice of surgical approach remains a subject of debate. The uniatrial approach offers a simpler and less invasive route but may provide limited visualization, especially in cases of bilateral or septal involvement. Conversely, the biatrial approach allows for superior exposure of both atria, facilitating thorough inspection and complete resection, particularly when synchronous or septal tumors are present. However, this technique may be associated with a higher incidence of postoperative arrhythmias and bleeding due to extensive atrial incisions and proximity to the conduction system [13,39]. In cases with septal invasion or biatrial extension, alternative and modified surgical techniques have been described, including the superior septal approach (SSA), inverted-T biatrial incision, and the double-patch technique [40,41]. In our study, the majority of patients underwent surgical excision through the right atrial approach (44.5%). A biatrial approach was performed in 14 cases (16.8%), while the left atrial approach was used in 4 patients (4.8%). Two patients experienced tumor recurrence, one after a right atrial approach and one following a biatrial approach. Four patients required permanent pacemaker implantation postoperatively due to sinus node dysfunction or conduction abnormalities. The median sternotomy remains the most commonly used surgical approach, performed in 49 patients (59.0%) in our study. In addition, one patient (1.2%) underwent surgery via ministernotomy, one (1.2%) through thoracotomy, and two patients (2.4%) through a minimally invasive endoscopic approach. Minimally invasive and endoscopic techniques have been developed as feasible alternatives to conventional sternotomy, offering superior visualization and reduced surgical trauma. However, they are associated with a longer learning curve, higher equipment costs, and a non-negligible risk of complications [42,43,44]. The differential diagnosis of cardiac myxomas includes benign and malignant tumors and intracardiac thrombi. Differentiation from thrombus is essential, as surgical outcomes are better in myxomas. Cardiac MRI provides superior tissue characterization: myxomas are iso/hypointense on T1, hyperintense on T2, and show delayed enhancement due to vascularity, whereas avascular thrombi remain hypointense on delayed images [45,46]. Cardiac myxomas may present with variable histologic patterns, one of which is the glandular subtype, accounting for less than 5% of cases and potentially mimicking mucin-secreting adenocarcinoma, making accurate diagnosis crucial. Macroscopically, myxomas may be solid, with smooth and firm borders, or papillary, with an irregular and friable surface. Microscopically, they exhibit a myxoid stroma containing round or spindle cells, occasional multinucleated cells near small vessels, and polygonal surface cells arranged in a single layer. Right-sided tumors are more frequently papillary, whereas left atrial septal myxomas are typically solid [5,47].

## 6. Conclusions

Bilateral and biatrial atrial myxomas represent exceptionally rare cardiac tumors. In many cases, the initial clinical manifestations are related to cerebral or peripheral embolic events or pulmonary thromboembolism, emphasizing the need for early detection. Screening echocardiography is particularly valuable in patients with a familial predisposition, allowing for the identification of asymptomatic cases.

Transthoracic and transesophageal echocardiography, combined with a meticulous evaluation of all cardiac chambers, are essential not only for diagnosis but also for preventing recurrence by identifying small or residual tumor fragments.

The minimally invasive endoscopic approach has proven to be a safe and effective surgical alternative for biatrial myxomas, offering excellent visualization and enabling a comprehensive inspection of the cardiac cavities.

For the differentiation between bilateral and biatrial forms, it is important to identify whether there is an interatrial communication and if the tumors arise from a common origin. These features can be assessed through imaging studies, intraoperative findings, or histopathologic examination, particularly in cases with mirror-like attachment sites, where the distinction may be more subtle.

## 7. Limitations

This review has several limitations directly related to the characteristics of the available literature. The analysis is based entirely on published case reports and case series, which contain heterogeneous and sometimes incomplete information. Due to the extreme rarity of this condition and the reliance on published cases from specialized centers, the generalizability of the findings to the broader population is limited.

## Data Availability

The original contributions presented in this study are included in the article/Supplementary Materials. Further inquiries can be directed to the corresponding author.

## Supplementary Materials

The following supporting information can be downloaded at: https://www.mdpi.com/article/10.3390/medsci13040294/s1, Table S1: Summary of all identified articles, including authors and year of publication.
